# Unskilled and unaware: second-order judgments increase with miscalibration for low performers

**DOI:** 10.3389/fpsyg.2024.1252520

**Published:** 2024-06-17

**Authors:** Mariana Veiga Chetto Coutinho, Justin Thomas, Imani Fredricks-Lowman, Shama Alkaabi, Justin J. Couchman

**Affiliations:** ^1^Department of Cognitive Sciences, College of Humanities and Social Sciences, United Arab Emirates University (UAEU), Al Ain, United Arab Emirates; ^2^The King Abdulaziz Center for World Culture, Dhahran, Saudi Arabia; ^3^Center for Teaching and Learning, Florida Memorial University, Miami Gardens, FL, United States; ^4^Department of Psychology, College of Natural and Health Sciences, Zayed University, Abu Dhabi, United Arab Emirates; ^5^Department of Psychology, Albright College, Reading, PA, United States

**Keywords:** overconfidence, second-order judgments, calibration accuracy, unskilled-unaware effect, self-insight, exam performance, Dunning-Kruger effect

## Abstract

Overestimation and miscalibration increase with a decrease in performance. This finding has been attributed to a common factor: participants’ knowledge and skills about the task performed. Researchers proposed that the same knowledge and skills needed for performing well in a test are also required for accurately evaluating one’s performance. Thus, when people lack knowledge about a topic they are tested on, they perform poorly and do not know they did so. This is a compelling explanation for why low performers overestimate themselves, but such increases in overconfidence can also be due to statistical artifacts. Therefore, whether overestimation indicates lack of awareness is debatable, and additional studies are needed to clarify this issue. The present study addressed this problem by investigating the extent to which students at different levels of performance know that their self-estimates are biased. We asked 653 college students to estimate their performance in an exam and subsequently rate how confident they were that their self-estimates were accurate. The latter judgment is known as second-order judgments (SOJs) because it is a judgment of a metacognitive judgment. We then looked at whether miscalibration predicts SOJs per quartile. The findings showed that the relationship between miscalibration and SOJs was negative for high performers and positive for low performers. Specifically, for low performers, the less calibrated their self-estimates were the more confident they were in their accuracy. This finding supports the claim that awareness of what one knows and does not know depends in part on how much one knows.

## 1 Introduction

When people are asked to evaluate their own performance in a test, their judgments are often not in line with their performance (Dunning et al., 2003, 2004; Couchman et al., 2016; De Bruin et al., 2017; Sanchez and Dunning, 2018; Coutinho et al., 2020). They tend to overestimate themselves and those with the lowest test scores tend to show greater levels of miscalibration (e.g., Kruger and Dunning, 1999; Ehrlinger et al., 2008; Moore and Healy, 2008; Pennycook et al., 2017; Coutinho et al., 2021). Because miscalibration is more pronounced among those who perform poorly in a test, researchers have referred to this finding as the unskilled-unaware effect (Kruger and Dunning, 1999; Dunning et al., 2003). However, whether higher levels of miscalibration indicate lack of awareness about what one knows or does not know is debatable.

Studies looking at overestimation among participants at different levels of performance tend to use a similar method to analyze the data. Researchers first separate the data into quartiles based on participants’ actual test performance, and then compare actual performance to participants’ estimated performance per quartile. This analysis yields a consistent pattern. Overestimation and miscalibration are greater among students in the bottom quartile, and decrease as performance increases. But when performance is very high, a shift to underestimation is observed. Because this finding was first reported by Kruger and Dunning (1999), it is also known as the Dunning-Kruger (DK) effect. The DK effect has since been shown by numerous studies with various tasks including reasoning tests (Pennycook et al., 2017; Coutinho et al., 2021), knowledge-based tests like geography (Ehrlinger and Dunning, 2003), skill-based tests of driving (Marottoli and Richardson, 1998), card gaming (Simons, 2013), sport coaching (Sullivan et al., 2018), and ability-based tests of emotional intelligence (Sheldon et al., 2014).

The primary interpretation of these findings is that low performers overestimate because they are unaware about how poorly they performed due to their own lack of knowledge. Kruger and Dunning (1999) posited that when people have limited knowledge or skills in a domain they are tested in, the lack of knowledge carries a double burden because it prevents people from performing well in the task and from knowing how poorly they did. They proposed that the same knowledge and skills required to do well in a task are also needed for knowing how well one did.

Although the dominant account of these findings is compelling, this is not the only one. Researchers have proposed that this finding could be explained by the hard-easy effect (Juslin et al., 2000), regression to the mean (Feld et al., 2017), regression better than average (BTA) approach (Krueger and Mueller, 2002; Burson et al., 2006), and boundary restrictions (Magnus and Peresetsky, 2022). The hard-easy effect is a tendency that people have of overestimating themselves when a task is difficult and underestimating themselves in easy tasks. In the DK effect, although objective task difficulty does not vary among participants, subjective difficulty varies. The test is difficult for those with low scores but easy for those with high scores. So, according to this account, subjective test difficulty is what drives overestimation. Researchers have also proposed that BTA- the tendency for people to perceive their skills better than average combined with regression to the mean explain the DK effect. Lastly, some researchers have stated that the DK effect occurs because the data is bounded. Based on this view, students in the bottom quartile overestimate because their average performance is relatively low, and performance is bounded at 0. This means that there is great room for overestimation but little for underestimation. For example, if average performance is 30 out of 100, participants can underestimate by 30 points, but they can overestimate by 70 points. So, the likelihood that they will overestimate is higher. The opposite pattern happens for high performers who have little room for overestimation since the maximum score is 100, and therefore are more likely to underestimate themselves.

The DK effect is an observable phenomenon demonstrated by numerous studies. But there are conflicting explanations of it that are supported by empirical evidence. These conflicting accounts are partly due to how calibration accuracy is measured and then compared across performance quartiles. This method is subjected to statistical artifacts like boundary restrictions that are difficult to avoid. Therefore, we argue that using self-estimates and calibration accuracy as indicators of awareness is not ideal. An alternative and more conservative option would be to ask participants to judge the accuracy of their own estimates of performance. That is, ask them to make second-order judgments, which are metacognitive judgments of metacognitive judgments. By doing so and comparing their SOJs with calibration accuracy (estimates of performance minus actual performance), we can get an index of whether they have some insight that their self-estimates are biased. This would be a more conservative indicator of awareness than calibration accuracy *per se*. According to Kruger and Dunning’s (1999) account, low performers are expected to be quite confident that their self-estimates are accurate because they do not have the means to properly evaluate themselves.

To date a few studies have utilized SOJs to evaluate awareness among students at different performance levels (Miller and Geraci, 2011; Händel and Fritzsche, 2016; Fritzsche et al., 2018; Nederhand et al., 2020). For instance, Miller and Geraci (2011) asked participants to predict their performance in an exam and then rate how confident they were that their self-estimates were well calibrated using a 5-point Likert scale. The results showed that low performing students overestimated how much they got in the test, however, they were less confident that their self-estimates were close to their actual test score than were high performing students. Similar results were found by Händel and Fritzsche (2016) using local metacognitive judgments (judgments for single questions) and postdictions (estimates of performance after completing a test) followed by SOJs. In both studies, low performing students overestimated their performance but their SOJs were lower than high performing participants. These findings thus indicate that low performing students are not as confident about the accuracy of their judgments as high performers, but this does not suggest that they have some level of awareness that their judgments are not well calibrated. Fritzsche et al. (2018) provided evidence for that by reanalyzing the data of Händel and Fritzsche and demonstrating that low performers’ SOJs did not change significantly as a function of how well calibrated their self-estimates were. To know whether low performers have some level of awareness, we need to look at the relationship between SOJs and miscalibration of self-estimates. Nederhand et al. (2020) did exactly that with a sample of college students and another of high school. But in contrast to previous studies, low performers did not show greater levels of miscalibration in terms of overconfidence. In fact, low performers were more accurate in their self-assessments than high performers. Additionally, they found a negative correlation between SOJs and miscalibration in the sample of university students. The more miscalibrated participants’ self-estimates were, the less confidence they had on them. This relationship suggests that participants in the sample were somewhat aware about the accuracy of their self-estimates. A question that arises from this finding is whether such a relationship between miscalibration and SOJs would differ between low and high performers. Based on Kruger and Dunning’s (1999) account of the DK effect, low performers’ SOJs would not increase with a decrease in miscalibration.

The purpose of the present study is to investigate the extent to which students at different levels of performance know that their self-estimates are biased. To do so, university students after completing a regular course exam estimated their performance in the exam, and then rated how confident they were that their self-estimates of performance were accurate by making a SOJ. We then looked at the relationship between SOJs and miscalibration (| estimated performance – actual performance|) among low and high performers separately. It was hypothesized that high levels of miscalibration would be associated with lower SOJs for students who performed well in the exam replicating Nederhand et al.’s (2020) findings. Regarding low performers, if indeed they are not aware of how well they did in the test as proposed by Kruger and Dunning’s (1999), then it is expected that the relationship between miscalibration and SOJs would be positive or nonexistent.

## 2 Methods

### 2.1 Participants

First-year undergraduate students enrolled in a course titled “Living Science: Health and Environment” at the participating university were invited to participate in the study. Six hundred and fifty-three undergraduate students from 30 different sections of the course volunteered to participate in the study. Students had to be at least 18 years-old to be eligible to participate in the study. All participants were from the United Arab Emirates, and 89% of them were females. The mean age of participants was 20 (*SD* = 2.08). They were bilingual in English and Arabic. All participants gave written informed consent prior to the study’s commencement.

### 2.2 Materials

**Self-Assessment:** At the end of a summative quiz, students were asked to estimate their score in the quiz out of 100. Subsequently, they were asked to evaluate the accuracy of their estimates by providing a SOJ using a 5-rating scale; from “I am not at all confident in it” to “I am very confident about it”. Participants added their responses to a piece of paper. The quiz took place on campus and included 20 multiple-choice questions. The quiz was about “Demography and Population Health” and counted for 10% of the total course grade.

**Design and Procedure:** The research ethics committee at Zayed University approved the study (ZU19_103_F). A week prior to the administration of the second quiz of the course “Living Sciences: Health and Environment”, students were informed about the study by their teacher in class and also through email. On the day of the quiz, students were given an informed consent form and briefed about what was expected from them if they chose to participate in the study. Students were informed that they could withdraw from the study at any time and that their responses would not affect their grade on the exam. Those who wished to participate were also given a demographic form with questions about age and gender. Upon completion of the exam, students who consented to participate stayed in the classroom and were asked to fill out a form with a postdiction question and a SOJ question. The questions were presented in English and Arabic.

### 2.3 Data analysis

Overestimation (or underestimation) was calculated by subtracting actual score from estimated score. We did that for every single participant. Positive values correspond to overestimation and negative to underestimation. In order to examine the degree of miscalibration, we calculated the absolute value of the difference between estimated performance and actual performance using the formula: |estimated performance – actual performance|. A score of 0 means no miscalibration, or perfect calibration accuracy.

## 3 Results

### 3.1 Overestimation

Participants overestimated their scores on the exam. The mean estimated score was 78.5 (*SD* = 11.5), whereas the average actual score was 67.8 (*SD* = 16), *t* (652) = 19, *p* < 0.001, *d* = 0.74. The correlation between estimated and actual scores was moderate positive, *r* (651) = 0.49, *p* < 0.001, indicating that participants’ metacognitive judgments predicted performance in the correct direction—that is, higher self-estimates were associated with higher exam scores.

### 3.2 Dunning-Kruger effect

**FIGURE 1:**
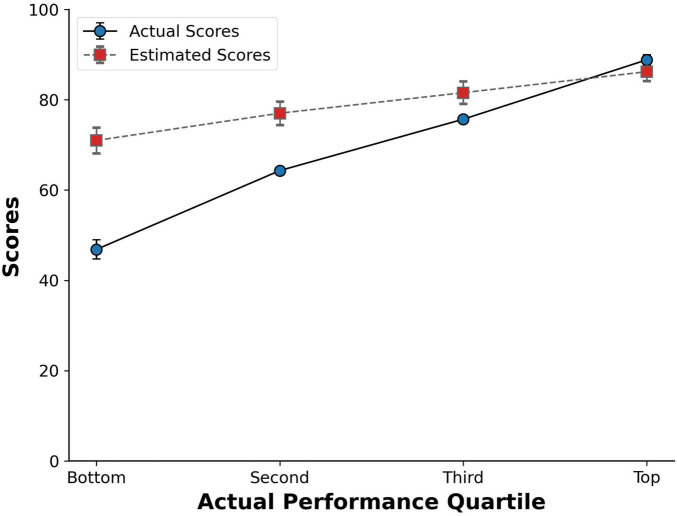
Estimated and actual scores for each performance quartile. Errors bars indicate standard errors.

To explore the accuracy of estimated scores across different levels of objective performance (bottom, second, third, and top quartile), a quartile-split based on actual performance in the exam was used. Size of quartiles ranged from 133 to 180 students. Mean of actual scores for each quartile and its sample size are shown on Table 1. As a manipulation check, a quartile Analysis of Variance (ANOVA) on actual performance was performed. Actual scores differed significantly across quartiles, *p* < 0.001. To evaluate whether the difference between estimated and actual scores varied across students at different quartiles, we conducted a mixed ANOVA with quartile as the independent variable and estimated and actual scores as dependent variables. The analysis yielded an interaction between quartile, estimated and actual scores, *F* (3,649) = 169.28, *p* < 0.001, η2 = 0.439. This means that the difference between estimated and actual scores differed across quartiles. A Tukey Honest Significant Difference (HSD) post-hoc test indicated that the difference between estimated and actual scores was significantly higher for bottom performers than for second (*p* < 0.001), third (*p* < 0.001) and upper quartile (*p* < 0.001) groups. As Figure 1 shows, this difference decreased with an increase in performance quartile. Students in the bottom quartile (*M* = 46.8, *SD* = 8.4) on average estimated that they had a score of 71.0 (*SD* = 11.4), overestimating their performance by 24.1 points, *t* (173) = 4.13, *p* < 0.001, *d* = 1.84. Similarly, students in the lower and upper middle quartile overestimated their performance by 12.7 (*t* [165] = 15.0, *p* < 0.001, *d* = 1.17) and 5.9 points (*t* [179] = 7.8, *p* < 0.001, *d* = 0.58). Whereas high performers underestimated their performance by only 2.6 points, *t* (132) = 3.7, *p* < 0.001, *d* = 0.32. We also compared miscalibration between participants in the top and bottom quartiles to know whether the absolute difference between self-estimates and actual scores differed between the two quartiles. Notably, miscalibration was greater for lower (*M* = 24.54, *SD* = 12.32) than high performers (*M* = 6.9, *SD* = 5.2), *t* (305) = 15.48, *p* < 0.001.

**TABLE 1:** Means, SDs and sample size per quartile.

	Quartile 1 (*N* = 174)	Quartile 2 (*N* = 166)	Quartile 3 (*N* = 180)	Quartile 4 (*N* = 133)
Actual scores	46.9 (8.4)	64.3 (3.1)	75.7 (3.3)	88.8 (4.6)
Estimated scores	71.0 (11.4)	77.0 (10.4)	81.6 (10.0)	86.2 (8.3)
SOJs	3.2 (0.90)	3.4 (0.83)	3.7 (0.93)	3.9 (0.81)
Overconfidence	24.1 (13.1)	12.7 (10.89)	5.9 (10.12)	−2.63 (8.25)
Miscalibration	24.5 (12.32)	14.5 (8.42)	9.4 (6.93)	6.9 (5.2)

### 3.3 Self-estimates across quartiles

Using an ANOVA with quartile as the independent variable and estimated scores as the dependent variable, we observed that estimated scores varied significantly across quartile groups, *F* (3,649) = 63.8, *p* < 0.001, η2 = 0.228. This analysis was followed by Tukey HSD post-hoc test, which indicated that all pairwise comparisons were significantly different, *p* < 0.001. The lower the quartile, the lower their estimates of their performance.

### 3.4 The relationship between SOJs and miscalibration

**FIGURE 2:**
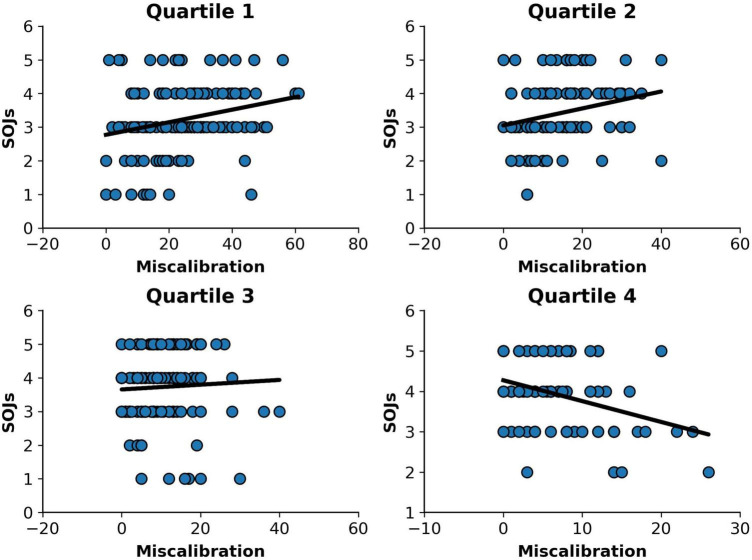
Relationship between miscalibration and SOJs per performance quartile.

To examine differences in metacognitive awareness across participants at different levels of performance, we regressed second-order judgments on miscalibration for each quartile separately (see Figure 2). For low performers, higher levels of miscalibration predicted an increase in SOJs, *b* = 0.019, se = 0.005, *t* (172) = 3.43, *p* < 0.001, R^2^ = 0.064, adjusted R^2^ = 0.059, *p* < 0.001. The same pattern was observed for participants in the second quartile, *b* = 0.064, se = 0.023, *t* (164) = 2.8, *p* = 0.006, R^2^ = 0.046, adjusted R^2^ = 0.040, *p* = 0.006. This shows that SOJs increases with an increase in miscalibration. In other words, the higher the discrepancy between estimated score and actual scores, the greater participants’ confidence that their estimated scores were close to their actual scores. As expected, high performers showed the opposite pattern. High levels of miscalibration predicted a decreased in SOJs, *b* = −0.052, se = 0.013, *t* (131) = 4.04, *p* < 0.001, R^2^ = 0.111, adjusted R^2^ = 0.104, *p* < 0.001. High performers gave lower SOJs when their self-estimates were farther from actual scores—that is, less well calibrated. No relationship between miscalibration and SOJs was found for the third quartile alone.

### 3.5 The relationship between SOJs and self-estimates, and SOJs and exam scores

To further explore metacognitive awareness across participants at different levels of performance, we regressed second-order judgments on self-estimates for each quartile separately. Higher SOJs were associated with higher self-estimates for participants in the first quartile, *b* = 0.028, se = 0.006, *t* (172) = 4.81, *p* < 0.001, R^2^ = 0.119, adjusted R^2^ = 0.114, *p* < 0.001; second quartile, *b* = 0.045, se = 0.019, *t* (164) = 2.44, *p* = 0.016, R^2^ = 0.035, adjusted R^2^ = 0.29, *p* = 0.016; third quartile, *b* = 0.05, se = 0.006, *t* (178) = 8.506, *p* < 0.001, R^2^ = 0.289, adjusted R^2^ = 0.285, *p* < 0.01; and fourth quartile, *b* = 0.065, se = 0.006, *t* (131) = 10.23, *p* < 0.001, R^2^ = 0.444, adjusted R^2^ = 0.440, *p* < 0.001. We also regressed second-order judgments on exam scores and found no significant relationship between the two variables for participants in the first (*p* = 0.332), second (*p* = 0.434) and third quartile (*p* = 0.171), respectively. However, for the fourth quartile, exam scores did predict SOJs, *b* = 0.045, se = 0.015, *t* (131) = 3.047, *p* = 0.003, R^2^ = 0.066, adjusted R^2^ = 0.059, *p* = 0.003, but not as well as self-estimates did. The positive association between SOJs and self-estimates may be an indication that participants rely on similar mechanisms when making both judgments. Zero-order correlations are shown in Supplementary Table 1.

### 3.6 SOJs

A quartile ANOVA on SOJs was conducted to evaluate whether low performers differed from more proficient peers on how confident they were that their estimated scores were well calibrated. The analysis showed that confidence judgments varied significantly across quartile groups, *F* (3,649) = 19.1, *p* < 0.001, η2 = 0.081 (see Table 1). Pairwise comparisons using Tukey HSD indicated that low performers were significantly less confident on their estimates than upper middle (*p* < 0.001) and high performers (*p* < 0.001).

## 4 Discussion

In the present study, we investigated the extent to which low performers know that their self-estimates are inflated. To do so, we asked participants at different levels of performance to rate how confident they were that their self-estimates of performance were accurate. That is, they were asked to make SOJs. We then examined the relationship between miscalibration and SOJs for each quartile separately. It was expected that participants who are aware or somewhat aware about how well they did in the test would show greater confidence in their self-estimates when their self-estimates were closer to their actual test scores. This is exactly what we found for high performers. The less miscalibrated their self-estimates were, the more confident they were in them. However, the opposite pattern was found for low performers. Their SOJs increased with an increase in miscalibration.

The results of this study are in line with Kruger and Dunning’s (1999) interpretation of DK effect. They propose that when individuals are tested on a topic that they have limited knowledge in, they will have difficulty judging how well they did in the test because their ability to accurately evaluate what they know or do not know depends on the same knowledge and skills that led to poor performance. The current findings complement this account by showing that failings in metacognition are not easily detectable by the learner. But why is that the case? One possible explanation for this finding is that when individuals have limited knowledge about a topic they are tested on, they are less likely to have access to valid metacognitive cues—that is, cues that are predictive of performance. Therefore, they end up relying on surface-based (or fluency-based) cues such as retrieval fluency (the ease with which information comes to mind) or processing fluency. The problem is that cues like fluency predict performance only sometimes, mainly when changes in fluency coincide with variations in valid cues. For example: in a memory test, when increases in retrieval fluency is associated with increases in the strength of the memory signal (valid cue), retrieval fluency accurately predicts performance. Otherwise, it does not. In line with this, previous research has shown differences in cue utilization among students at different levels of performance (Thiede et al., 2010; Ackerman and Leiser, 2014; Gutierrez de Blume et al., 2017). For instance, Ackerman and Leiser (2014) demonstrated that low achievers – when regulating their learning of text—were more sensitive to unreliable, surface-level cues (e.g., ease of processing, readability, and specific vocabulary) than high achievers. Additionally, Thiede et al. (2010) showed that students who were at-risk readers were more likely to base their judgments of text-comprehension on surface-based cues. Conversely, competent readers relied on valid comprehension-based cues, such as the belief they can explain the text to another person. The results of these studies taken together suggest that when participants are less knowledgeable about the material they are tested on, they are more likely to rely on fluency-based cues and believe that such cues are good predictors of performance when in fact they are not.

We also found that estimated scores were a stronger predictor of SOJs than actual test scores for every quartile. This suggests that SOJs may depend on similar mechanisms as self-estimates. But it is likely that the cues that low performers rely on when making metacognitive and second-order judgments are not the same as these utilized by high performers and this is why for low performers confidence increases with miscalibration whereas for high performers confidence decreases with miscalibration.

Additionally, we compared SOJs between lower and higher performers, and we found differences between the two groups. Lower performers displayed less confidence in the accuracy of their estimates than more proficient peers. These findings are consistent with previous studies showing low performers’ general tendency to give lower confidence ratings (see Fritzsche et al., 2018). This finding could be also attributed to differences in cue utilization between low and high performing students. Although low performers rely on surface-based cues and are confident on them, such cues are unlikely to give rise to the same level of confidence as valid cues because they are unreliable.

### 4.1 Limitation and future directions

The present study has some limitations. First, participants were asked to estimate their performance after a real course exam. Exams are anxiogenic for many students and anxiety can influence the accuracy of one’s judgments. This can be corrected by using practice quizzes instead of real exams as a base for testing our hypothesis. At the same time, the present study design offers greater ecological validity. Second, there are other factors in addition to knowledge of the subject tested that could have contributed to the difference in miscalibration among students at different levels of perfomance, such as wishful thinking and academic self-efficacy that we did not measure. Future studies could look at such factors as potential mediators. Wishful thinking may have contributed to inflated self-estimates and SOJs in low performing students. Researchers could test that by asking participants to state their desired score in an exam prior to taking the exam, and then look at whether SOJs change as a function of how closely related desired and estimated scores are for low and high performers separately. Third, the number of males in the study was very small. Therefore, we do not know if the findings would generalize to males. Fourth, we did not test the effect of cues like processing fluency on metacognitive judgments. Additional studies could address this point by focusing on how various cues influence overestimation across low performers. This can be done by manipulating variables that purposely increase fluency, and assessing how low performers respond to such cues versus high performers using local metacognitive judgments—that is, judgments for single test items. One potential study could increase fluency in a memory test by, for example, increasing the size of stimuli, like words, which is known to increase processing fluency. The study could then test whether these who perform worse in the memory test are more likely to interpret this increase in fluency as an indicator of knowing by measuring the accuracy of their local metacognitive judgments. Future studies could also focus on designing and testing training programs to help low performers identify unreliable cues and how not to fall prey for them. This could be done through an interactive test where students are asked to rate their confidence for each question in a test, and then are prompted to reflect and report why they rated it the way they did. Once they given an their answer, feedback about the accuracy of their judgments would be provided. Through this training students may become aware that certain cues are unreliable indicators of performance, and therefore should not be relied on as much.

## 5 Conclusion

We provided evidence that an increase in miscalibration is associated with an increase in SOJs for low performers. This supports and complements the dual burden account of the DK effect. We suspect that this may be the result of how low performers interpret cues available during testing. Specifically, relying on cues that are fluency-based and not reflective of performance. It is likely that low performing students view increases in fluency as an indicator that their responses are correct, which then lead to inflated self-estimates and higher confidence in their self-estimates. The present study has theoretical implications to the literature on the DK effect, and practical implications to education. First, it shows that low performers lack awareness that their estimates are biased. Second, it suggests that proper calibration among low performers is unlikely to happen by itself. Hence, it is important that in school, students learn about these biases, its effects in learning and performance and what they can do to minimize its influences.

## Data availability statement

The datasets presented in this study can be found in online repositories. The names of the repository/repositories and accession number(s) can be found below: OSFHOME repository: https://osf.io/nqrhy/files/osfstorage/64a313b2a2a2f4128543694c.

## Ethics statement

The study involving human participants was reviewed and approved by Zayed University, UAE. The participants provided their written informed consent to participate in this study. Ethical clearance was obtained from Zayed University Research Ethics Committee (ZU19_103_F).

## Author contributions

MC contributed to conceptualization, review of the literature, design and methodology of the study, data analysis and interpretation, drafting of the manuscript, submission and review process. JT contributed to the literature review, data collection, and revision of the draft. IF-L contributed to recruiting participants, data collection, and data entry. SA contributed to the writing of the literature review and methods. JC contributed to drafting of the manuscript. All authors contributed to the article and approved the submitted version.
