# Supplementary figures and images for: Unskilled and unaware: second-order judgments increase with miscalibration for low performers
Source: Front Psychol. 2024 Jun 17;15:1252520. doi: 10.3389/fpsyg.2024.1252520 (PMC11215559; doi:10.3389/fpsyg.2024.1252520)

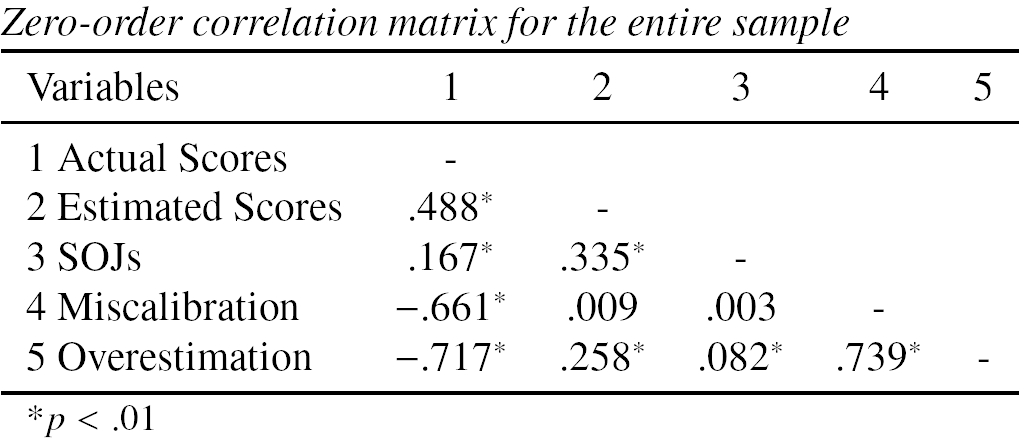

Supplement: Supplementary file 1 [file Image_1.JPEG]
